# Rectilinear Edge Selectivity Is Insufficient to Explain the Category Selectivity of the Parahippocampal Place Area

**DOI:** 10.3389/fnhum.2016.00137

**Published:** 2016-03-30

**Authors:** Peter B. Bryan, Joshua B. Julian, Russell A. Epstein

**Affiliations:** Department of Psychology, University of PennsylvaniaPhiladelphia, PA, USA

**Keywords:** fMRI, scene perception, neural specialization, vision, ventral stream

## Abstract

The parahippocampal place area (PPA) is one of several brain regions that respond more strongly to scenes than to non-scene items such as objects and faces. The mechanism underlying this scene-preferential response remains unclear. One possibility is that the PPA is tuned to low-level stimulus features that are found more often in scenes than in less-preferred stimuli. Supporting this view, Nasr et al. (2014) recently observed that some of the stimuli that are known to strongly activate the PPA contain a large number of rectilinear edges. They further demonstrated that PPA response is modulated by rectilinearity for a range of non-scene images. Motivated by these results, we tested whether rectilinearity suffices to explain PPA selectivity for scenes. In the first experiment, we replicated the previous finding of modulation by rectilinearity in the PPA for arrays of 2-d shapes. However, two further experiments failed to find a rectilinearity effect for faces or scenes: high-rectilinearity faces and scenes did not activate the PPA any more strongly than low-rectilinearity faces and scenes. Moreover, the categorical advantage for scenes vs. faces was maintained in the PPA and two other scene-selective regions—the retrosplenial complex (RSC) and occipital place area (OPA)—when rectilinearity was matched between stimulus sets. We conclude that selectivity for scenes in the PPA cannot be explained by a preference for low-level rectilinear edges.

## Introduction

Functional magnetic resonance imaging (fMRI) studies have identified several brain regions that respond preferentially to visual scenes. For example, a region in ventral temporal cortex known as the parahippocampal place area (PPA) responds more strongly when people view scenes (e.g., landscapes, cityscapes, rooms) than when they view isolated single objects or faces (Aguirre et al., 1998; Epstein and Kanwisher, 1998). Although the robustness of this scene-preferential response is well-established, the mechanism behind it is not entirely understood. The standard explanation is that the PPA selectivity reflects tuning to a high-level stimulus category such as “scene”, “landmark”, or “place” (Epstein, 2005; Downing et al., 2006). However, an alternative possibility is that the PPA is tuned for low-level features that are more commonly found in scenes than in other non-preferred stimulus categories.

Recent work has provided some support for the low-level feature explanation by demonstrating response biases in the PPA that relate to the distribution of low-level features. For example, the PPA responds more strongly to high spatial frequency stimuli than low spatial frequency stimuli (Rajimehr et al., 2011; Zeidman et al., 2012; Kauffmann et al., 2015; Watson et al., 2016) and more strongly to images with edges at cardinal orientations (vertical, horizontal) than to images with edges at non-cardinal orientations (Nasr and Tootell, 2012; Lescroart et al., 2015). Moreover, in an intriguing recent study, Nasr et al. (2014) report that the PPA is sensitive to the presence of rectilinear edges: it responds more strongly to stimuli with many right angles than to stimuli with few right angles, even when the stimuli are basic shapes without any high-level semantic content. These authors further report that this preference for right angles is at least as large as the PPA preference for scenes, and they note the interesting fact that many stimuli that strongly activated the PPA in previous studies had a large quantity of right angles. They speculate that scene-selectivity in the ventral visual stream might be explained by a sensitivity to rectilinear edges—an idea that has some plausibility given the ubiquitous nature of rectilinear junctions in modern built environments. We will henceforth refer to the idea that rectilinearity sensitivity might explain PPA scene selectivity as the “rectilinearity hypothesis.”

Although these results are suggestive, the presence of low-level feature biases in a region does not preclude the possibility that it might also encode high-level category information that is tolerant to transformations of those low-level features. As an example, the PPA exhibits retinotopic organization (Levy et al., 2001, 2004; Arcaro et al., 2009; Silson et al., 2015), but it also represents scene identity in a manner that is invariant to retinotopic location (MacEvoy and Epstein, 2007; Golomb and Kanwisher, 2011). Nor is it clear that these low-level biases are sufficient to explain all aspects of regional tuning. For example, the PPA response to objects and buildings is modulated by their navigational history, an effect that cannot be explained in terms of the low-level visual features of the objects and buildings (Janzen and van Turennout, 2004; Schinazi and Epstein, 2010). Moreover, although Nasr and colleagues established a rectilinearity effect for single objects, arrays of objects, and arrays of geometric shapes, they did not examine the effect of rectilinearity on PPA response to naturalistic scenes. Thus, it remains possible that category selectivity rather than low-level feature selectivity best characterizes the response properties of the PPA.

The present study addresses this issue. We present results from three experiments that aimed to determine whether rectilinearity suffices to explain the scene-selectivity of the PPA. The first experiment attempted to replicate Nasr and colleagues’ finding of a rectilinearity bias for basic shapes in the PPA. The second experiment examined whether a similar rectilinearity bias could be found for naturalistic stimuli (i.e., scenes and faces), and tested whether the “categorical” difference between scenes and faces in the PPA would be maintained when rectilinearity was matched between the two stimulus classes. The third experiment again tested for a rectilinearity bias in naturalistic stimuli, by using faces and scenes with artificially enhanced or degraded rectilinearity. To anticipate, our results show that the PPA is indeed sensitive to rectilinearity for arrays of 2-d shapes, but rectilinearity does not suffice to explain scene-sensitivity of the PPA.

## Materials and Methods

### Participants

Participants were recruited from the University of Pennsylvania community to participate in one of three experiments (Experiment 1: *n* = 8, 4 female, age range: 21–38; Experiment 2: *n* = 8, 3 female, age range 20–38; Experiment 3: *n* = 15, 7 female, age range: 20–38). Five subjects that participated in Experiment 1 also participated in Experiment 2 (separated by around 3 months). All subjects that participated in Experiment 1 also participated in Experiment 3 during the same testing session, with Experiment 3 preceding Experiment 1. Subjects had normal or corrected-normal vision and had radiologically normal brains, without history of neuropsychological disorder. All participants provided written consent according to procedures approved by the University of Pennsylvania institutional review board.

### MRI Acquisition

Scanning was performed at the Hospital of the University of Pennsylvania using a 3T Siemens Trio scanner equipped with a 32-channel head coil. High-resolution T1-weighted images for anatomical localization were acquired using a three-dimensional magnetization-prepared rapid acquisition gradient echo pulse sequence [repetition time (TR), 1620 ms; echo time (TE), 3.09 ms; inversion time (TI), 950 ms; voxel size, 1 × 1 × 1 mm; matrix size, 192 × 256 × 160]. T2*-weighted images sensitive to blood oxygenation level-dependent (BOLD) contrasts were acquired using a gradient echo, echoplanar pulse sequence [TR, 3000 ms; TE, 30 ms; flip angle 90°; voxel size, 3 × 3 × 3 mm; field of view (FOV), 192; matrix size, 64 × 64 × 44]. Visual stimuli were displayed by rear-projecting them onto a Mylar screen at 1024 × 768 pixel resolution with an Epson 8100 3-LCD projector equipped with a Buhl long-throw lens. Subjects viewed stimuli through a mirror attached to the head coil.

### General Design and Procedure

Each experiment consisted of two 5 min 25 s fMRI scan runs in which subjects viewed stimuli from four conditions that were chosen to test specific hypotheses about PPA function (see “Stimuli” Section). Scan runs were divided into sixteen 15 s blocks; in each, subjects viewed 15 stimuli from the same condition presented one at a time for 600 ms each followed by a 400 ms interstimulus interval. Stimuli had a visual extent of approximately 13 × 13 degrees and were presented on a gray background. The experimental blocks were interspersed with five 15 s fixation blocks in which a black fixation cross was presented at the middle of a uniform gray screen. Subject attention was maintained by asking them to perform a one-back image repetition detection task during the experimental blocks. Stimulus repetitions occurred twice per block; thus, there were 52 unique stimuli per condition.

Following the experimental runs, subjects completed two functional localizer runs in which they viewed scenes, objects, faces, and scrambled objects in separate blocks. Data from these runs were used to identify the location of the PPA and other scene-selective regions. These runs had the same length, design, timing, and task as the main experimental runs.

### Stimuli

#### Experiment 1

**Figure 1:**
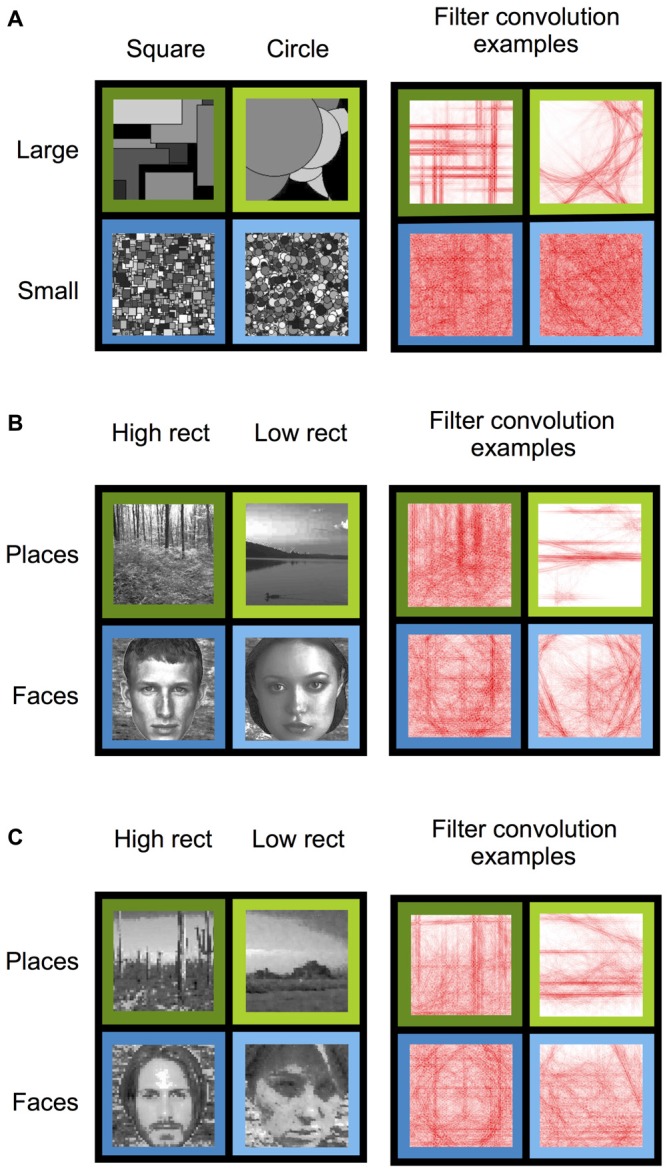
**Stimulus conditions (left) and example right angle convolution intensities (right) for Experiments 1–3. (A)** Experiment 1 stimuli consisted of squares (high-rectilinearity) and circles (low-rectilinearity) that were either large or small in size. **(B)** Experiment 2 stimuli consisted of naturalistic high- and low- rectilinearity scene and face images. **(C)** Experiment 3 stimuli consisted of pixilated (high-rectilinearity) and pointillized (low-rectilinearity) scene and face images.

To replicate Nasr and colleagues finding of an effect of rectilinearity on PPA response during viewing of geometric shapes, subjects were presented with arrays of computer-generated 2D squares (high-rectilinearity) or circles (low-rectilinearity; Figure 1A). Because it is unknown whether the PPA rectilinearity bias depends on the spatial extent of the rectilinear edges, the squares and circles in the two shape arrays were generated at two different sizes (large and small; Figure 1A). Widths of squares and diameters of circles were ten times larger, on average, in the large shape conditions than the small shape conditions. Fifty two unique images were generated per condition. Each individual shape in an array in a given image was randomly assigned a gray-scale fill. Stimulus conditions were matched on mean luminance and contrast using the SHINE toolbox (Willenbockel et al., 2010).

Rectilinearity for each condition was calculated using the methods outlined in Nasr et al. (2014) and clarified through personal correspondence with the authors. In brief, right angle wavelet filters were first constructed with an algorithm originally used to generate curved “banana” filters (Krüger et al., 1996). Rather than using a square root function to produce curved filters, however, an absolute value function was used to produce angled filters. Wavelets were constructed at four different spatial scales (1/5,1/9,1/15, and 1/27 cycles per pixel) and 16 different orientations (22.5–360° in 22.5° steps) at a size of 300 pixels by 300 pixels. Edges in the images were then extracted using Canny edge detection at a threshold of 0.2 and each filter was individually convolved with the edge map. Intensities from the resultant convolved matrix were averaged across edge points and orientations to generate orientation-invariant wavelet coefficients. These coefficients were then normalized within spatial scale across the image set for each experiment by subtracting the minimum value within spatial scale and dividing by the range. The final rectilinearity index for each image was determined by averaging these normalized coefficients across the four spatial scales. As expected, squares had significantly higher rectilinearity than circles (*t*_(206)_ = 5.54, *p* < 10^−7^).

#### Experiment 2

To test whether rectilinearity effects could be found for naturalistic stimuli, subjects were presented with grayscale images of faces and scenes that were grouped by rectilinearity (Figure 1B). Specifically, 52 high-rectilinearity scenes, 52 low-rectilinearity scenes, 52 high-rectilinearity faces, and 52 low-rectilinearity faces were chosen from a larger image set (377 faces; 543 scenes) based on their rectilinearity values. High-rectilinearity stimuli had, by design, significantly higher rectilinearity than low-rectilinearity stimuli (*t*_(206)_ = 27.92, *p* < 10^−72^). Crucially, high-rectilinearity faces and scenes were statistically matched (*t*_(102)_ = 1.53, *p* = 0.13) as were low-rectilinearity faces and scenes (*t*_(102)_ = 1.42, *p* = 0.16). Thus, response differences between faces and scenes could not be explained by differences in rectilinearity. To ensure that all stimuli had equal retinotopic extent, faces were displayed on a phase-scrambled variation of a single scene image, which was included in the rectilinearity calculation. Each stimulus condition was matched for mean luminance and contrast using the SHINE toolbox. All scene stimuli depicted natural outdoor scenes (e.g., forests, lakes, etc.).

#### Experiment 3

To further test whether the PPA and other scene regions are sensitive to the rectilinearity of naturalistic stimuli, we created a new set of grayscale images of natural faces and scenes, which had rectilinearity artificially enhanced or reduced. These images were pseudorandomly drawn from the same image set as in Experiment 2 and from the SUN image database (Xiao et al., 2010). The same images were presented to each participant. For each stimulus category, half of the images were decomposed into square pixels that were larger than the original pixels (high-rectilinearity) and half were decomposed into round points (low-rectilinearity). The result of these manipulations is to shift the perceptual salience of high spatial frequency rectilinearity up or down, respectively (Figure 1C). Pixelated images were divided into pixels aligned by row and column across the image. Pointillized images consisted of imbricated circles to cover the full image. Pixels and points had edges or diameters, respectively, of 6 pixels each at display resolution size. Pixilation and pointillization was executed using Pixelmator Software (v3.3.2, 2014). There were 52 unique images generated per condition (pixelated scene, pointillized scenes, pixelated faces, pointillized faces). Pixelated scenes had higher rectilinearity than pointillized scenes (*t*_(102)_ = 3.02, *p* < 0.01), and pixelated faces had higher rectilinearity than pointillized faces (*t*_(102)_ = 4.44, *p* < 0.0001). Further, rectilinearity was biased against the expected fMRI category effect in the PPA: pixelated faces had marginally greater rectilinearity than pixelated scenes (*t*_(102)_ = 1.88, *p* = 0.06) and pointillized faces had significantly greater rectilinearity than pointillized scenes (*t*_(102)_ = 3.26, *p* < 0.01). Each stimulus condition was matched for mean luminance and contrast using the SHINE toolbox.

### Data Analysis

Functional MR images for both the main experiments and functional localizer were preprocessed using the following steps. First, they were corrected for differences in slice timing by resampling slices in time to match the first slice of each volume. Second, they were corrected for subject motion by realigning to the first volume of the scan run using MCFLIRT (Jenkinson et al., 2002). Third, the timecourses for each voxel were high-pass filtered to remove low temporal frequency fluctuations in the BOLD signal that exceeded lengths of 100 s. Data from the functional localizer scan were smoothed with a 5 mm full-width at half-maximum (FWHM) Gaussian filter. Data from the experimental scans were smoothed with a 5 mm FWHM Gaussian filter for all region of interest analyses and 8 mm FWHM for all whole-brain group analyses.

We examined univariate responses within several regions of interest (ROI) known to be involved in visual processing. ROIs were defined individually for each subject using data from the functional localizer scans. In addition to the PPA, we defined ROIs for two other scene-responsive regions [retrosplenial complex (RSC) and occipital place area (OPA); Hasson et al., 2002; Bar and Aminoff, 2003; Dilks et al., 2013], and early visual cortex (EVC). The OPA, but not the RSC or EVC, has been previously reported to show a similar rectilinearity bias to PPA (Nasr et al., 2014). ROIs were defined using a contrast of scenes > objects for PPA, RSC, and OPA, and scrambled-objects > baseline for EVC, and they were further constrained by a group-based anatomical map of scene- or scrambled-object-selective activation derived from a large number (42) of localizer subjects that had been previously obtained in our lab (Julian et al., 2012). Specifically, each ROI was defined as the top 100 voxels in each hemisphere that exhibited the defining contrast and fell within the group-parcel mask for that ROI. The group-parcel mask for EVC was defined based on a scrambled-objects > intact-objects contrast. The voxels comprising each ROI did not need to be contiguous. This method ensured that all ROIs could be defined in both hemispheres in every subject and that all ROIs contained the same number of voxels. All ROIs were combined across hemispheres unless otherwise noted. All contrasts were performed in the native anatomical space for each subject and the group-parcel map was mapped into that space using a linear transformation.

We then used general linear models (GLMs) implemented in FSL^1^ to estimate the response of each voxel to the four experimental conditions for each experiment. Each condition was modeled as a boxcar function convolved with a canonical hemodynamic response function. To test for the effects of independent factors, we applied repeated-measures analysis of variances (ANOVAs) to the univariate responses within each ROI. Subsequent comparisons between individual conditions were based on paired-sampled *t-*tests. For tests of the rectilinearity hypothesis, significance was assessed using 1-tailed tests in the direction of the rectilinearity hypothesis (i.e., greater response to high- than low-rectilinearity conditions). For all other tests, significance was assessed using 2-tailed tests.

In addition to the ROI analyses, we also performed a whole-brain group analysis to test for effects of rectilinearity and category in Experiments 2 and 3 outside of our ROIs. For this analysis, data from those subjects who participated in both Experiments 2 and 3 were first combined via a within-subject fixed-effects analysis prior to the group analysis. To generate group-averaged maps, each individual participant’s functional maps were spatially transformed onto the averaged human brain using a spherical transformation in FreeSurfer^2^ (Fischl et al., 1999) and then averaged using random effects models in FSL.

In addition to univariate analyses, we also assessed whether there was information about rectilinearity and stimulus category represented in the multivoxel patterns of response in each ROI in Experiments 2 and 3. To do so, for each participant, we used GLMs to estimate the response pattern evoked by each stimulus condition separately for each of the two fMRI runs. Multivoxel pattern analyses (MVPA) were then performed through split-half pattern comparison (Haxby et al., 2001). Individual patterns were normalized prior to this computation by subtracting the grand mean pattern (i.e., the cocktail mean) for each half of the data. For each ROI, we then computed the correlation between the response patterns resulting from the same stimulus conditions and from different stimulus conditions. To test for coding of rectilinearity controlling for stimulus category, we computed a discrimination index that was the difference in the average correlation between the same rectilinearity condition and the corresponding different rectilinearity condition (i.e., [same rectilinearity, same category] − [different rectilinearity, same category]). This rectilinearity discrimination index was computed separately for scenes and faces. Likewise, to test for coding of stimulus category controlling for rectilinearity we computed a discrimination index that was the difference in the average correlation between the same category condition and the corresponding different category condition (i.e., [same category, same rectilinearity] − [different category, same rectilinearity]). This category discrimination index was computed separately for high and low rectilinearity stimulus conditions. To assess statistical significance, *t*-tests were used evaluate if the discrimination indices were greater than zero.

## Results

### Experiment 1: Does the PPA Respond More to High- than Low-Rectilinearity Shapes?

In our first experiment, we sought to replicate the PPA rectilinearity bias for simple shapes reported by Nasr et al. (2014). To do so, we scanned participants while they viewed arrays of computer-generated gray-scale squares (high-rectilinearity) and circles (low-rectilinearity) presented at two different sizes (small or large; Figure 1A). We then examined the fMRI response in each of the predefined ROIs.

**Figure 2:**
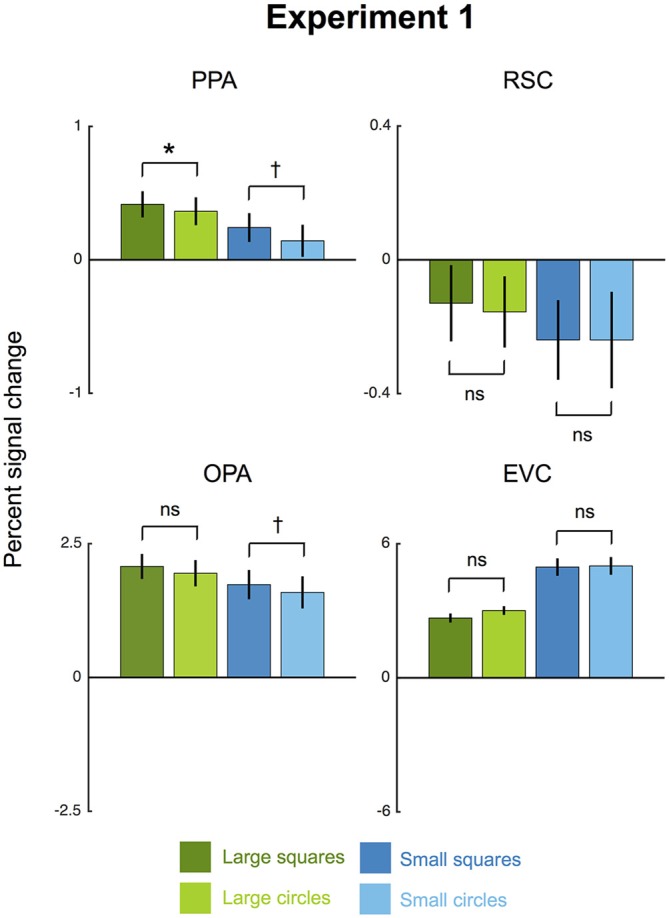
**Results for Experiment 1.** Average percent signal change (±1 SEM) to large and small squares (high-rectilinearity) and circles (low-rectilinearity) is shown for each region of interest (ROI) averaged across hemispheres. The parahippocampal place area (PPA) and occipital place area (OPA) both showed a significant main effect of shape, with greater overall responses to squares than circles. (^†^*p* < 0.07; *p < 0.05).

Consistent with the rectilinearity hypothesis, the PPA responded more strongly to arrays of squares than to arrays of circles (Figure 2). Confirming this observation, a 2 × 2 ANOVA with factors for shape (square vs. circle) and size (small vs. large) found a main effect of shape (*F*_(1,7)_ = 7.48, *p* < 0.05, $\eta_{p}^{2}$ = 0.52). The greater PPA response to squares than circles was significant for large shapes (*t*_(7)_ = 2.64, *p* < 0.05) and marginally significant for small shapes (*t*_(7)_ = 1.72, *p* = 0.07). Thus, our results replicate the basic finding of Nasr and colleagues that the PPA’s response to arrays of 2-d shapes is modulated by rectilinearity.

We also observed a significant effect of rectilinearity, with greater response to arrays of squares than to arrays of circles, in OPA (*F*_(1,7)_ = 10.37, *p* < 0.01, $\eta_{p}^{2}$ = 0.60). In contrast, there was no rectilinearity bias in RSC (*F*_(1,7)_ = 0.09, *p* = 0.77) or EVC (*F*_(1,7)_ = 2.94, *p* = 0.13). The fact that EVC responds equally to squares and circles suggests that the rectilinearity bias observed in PPA and OPA was not simply inherited from this region. The OPA showed a significantly greater rectilinearity effect than EVC (*F*_(1,7)_ = 8.64, *p* < 0.05, $\eta_{p}^{2}$ = 0.55) but the region-by-rectilinearity interaction between PPA and EVC fell short of significance (*F*_(1,7)_ = 3.89, *p* = 0.089).

Unexpectedly, we also observed size effects in the PPA, OPA, and RSC. All three scene regions responded significantly more to large than small shapes (PPA: *F*_(1,7)_ = 41.91, *p* < 0.01, $\eta_{p}^{2}$ = 0.86; RSC: *F*_(1,7)_ = 14.35, *p* < 0.01, $\eta_{p}^{2}$ = 0.59; OPA: *F*_(1,7)_ = 10.01, *p* < 0.01, $\eta_{p}^{2}$ = 0.67). The reason for this preference for large shapes is unclear. It may indicate a preference for larger objects (Konkle and Oliva, 2012), or it might be driven by uncontrolled variables such as spatial frequency or numerosity. Notably, EVC showed the opposite effect, responding more to small than large shapes (*F*_(1,7)_ = 72.97, *p* < 0.001, $\eta_{p}^{2}$ = 0.91). In no ROI was there was significant interaction between shape and size (PPA: *F*_(1,7)_ = 0.51, *p* = 0.50; RSC: *F*_(1,7)_ = 0.01, *p* = 0.92; OPA: *F*_(1,7)_ = 0.15, *p* = 0.71; EVC: *F*_(1,7)_ = 2.36, *p* = 0.17). Further, an additional 2 × 2 × 2 analysis with hemisphere as a factor found that the shape and size effects did not vary by hemisphere in any ROI (all *F*_(1,7)_s < 2.59, *p*s > 0.15).

### Experiment 2: Does the PPA Exhibit a Rectilinearity Effect for Naturalistic Stimuli (Scenes and Faces)?

After replicating the rectilinearity bias for shapes in the PPA, we next moved on to test whether there is a rectilinearity effect for naturalistic images, by scanning participants while they viewed images of high- and low-rectilinearity scenes and high- and low-rectilinearity faces (Figure 1B). Importantly, rectilinearity was matched between the scenes and faces; that is, the high-rectilinearity scenes and faces had a similar level of rectilinearity, as did the low-rectilinearity scenes and faces. Thus, our design not only allowed us to examine rectilinearity effects for scenes and faces, it also provided a strong test of the rectilinearity hypothesis. If the preferential response to scenes compared to faces in the PPA is due to the greater rectilinearity of scenes, then the “categorical” effect should be eliminated by rectilinear matching. On the other hand, if the PPA responds strongly to scenes in part because it is tuned to scenes as a category, then it should continue to exhibit a preferential response to scenes even after rectilinear matching.

**Figure 3:**
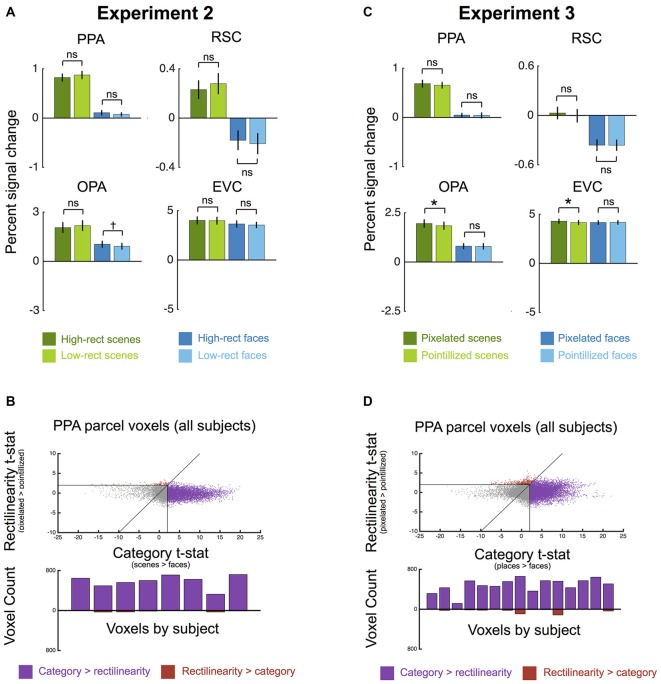
**Univariate results for Experiments 2 and 3. (A)** Experiment 2 average percent signal change (±1 SEM) to high-rectilinearity and low-rectilinearity scenes and faces in each ROI. No main effect of rectilinearity was observed in any ROI, although all ROIs showed a greater response to scenes than faces. **(B)** Experiment 2 comparison of rectilinearity-selectivity (high-rectilinearity > low-rectilinearity contrast *t*-statistic) and category-selectivity (scenes > faces contrast *t*-statistic) for all voxels in the group-defined PPA parcel for all participants (top row). Points that fall below the unity line are voxels with greater category-selectivity than rectilinearity-selectivity (shown in purple), and points that fall above the unity line are voxels with greater rectilinearity-selectivity than category-selectivity (show in red). Gray voxels were not significant (*p* < 0.05, uncorrected) for either contrast. Few voxels exhibited greater rectilinearity-selectivity than scene-selectivity. The bottom row shows a histogram of rectilinearity- and category-selective voxels in each participant. In all subjects, the number of category-selective voxels far exceeded the number of rectilinearity-selective voxels. **(C)** Experiment 3 average percent signal change (±1 SEM) to pixilated (high-rectilinearity) and pointillized (low-rectilinearity) scenes and faces in each ROI. No main effect of rectilinearity was observed in any ROI, although all ROIs showed a greater response to scenes than faces. **(D)** Experiment 3 comparison of rectilinearity-selectivity (pixilated > pointillized contrast *t*-statistic) and category-selectivity (scenes > faces contrast *t*-statistic) for all voxels in the group-defined PPA parcel for all participants (top row). As in Experiment 2, few voxels exhibited greater rectilinearity-selectivity than scene-selectivity. Further, in all participants the number of category-selective voxels again far exceeded the number of rectilinearity-selective voxels (bottom row). (^†^*p* < 0.07; *p < 0.05).

Results are plotted in Figure 3A. A 2 × 2 ANOVA with factors for category (scene vs. face) and rectilinearity (high vs. low) found a strong effect of category in the PPA (*F*_(1,7)_ = 153.65, *p* < 0.001, $\eta_{p}^{2}$ = 0.96), with greater response to scenes than to faces. Crucially, there was no main effect of rectilinearity (*F*_(1,7)_ = 0.49, *p* = 0.51). There was a significant interaction between category and rectilinearity (*F*_(1,7)_ = 11.26, *p* < 0.05, $\eta_{p}^{2}$ = 0.62); however, this interaction was driven by a *lower* response to high- than low-rectilinearity scenes (*t*_(7)_ = −3.77, *p* = 0.99) and the numerically converse effect for faces, though the rectilinearity effect for faces was not significant (*t*_(7)_ = 1.52, *p* = 0.17).

Results in the other two scene regions were similar to the PPA: both RSC and OPA responded significantly more to scenes than faces (RSC: *F*_(1,7)_ = 31.10, *p* < 0.01, $\eta_{p}^{2}$ = 0.82; OPA: *F*_(1,7)_ = 46.09, *p* < 0.001, $\eta_{p}^{2}$ = 0.87) but neither region exhibited a main effect of rectilinearity (RSC: *F*_(1,7)_ = 0.26, *p* = 0.63; OPA: *F*_(1,7)_ = 0, *p* = 0.99). Like PPA, OPA also showed a significant interaction between category and rectilinearity (*F*_(1,7)_ = 15.45, *p* < 0.01, $\eta_{p}^{2}$ = 0.69), with a lower response to high- than low-rectilinearity scenes (*t*_(7)_ = −3.10, *p* = 0.99) and a marginal rectilinearity effect for faces (*t*_(7)_ = 1.65, *p* = 0.06). There was no interaction between category and rectilinearity in RSC (*F*_(1,7)_ = 2.08, *p* = 0.19). Comparison of the three scene regions revealed no interaction between region and rectilinearity (*F*_(2,14)_ = 0.05, *p* = 0.95), but an interaction between region and category (*F*_(2,14)_ = 15.99, *p* < 0.001, $\eta_{p}^{2}$ = 0.70; scene-face response difference: OPA > PPA > RSC). Additional 2 × 2 × 2 ANOVAs with hemisphere as a factor found no significant interaction between hemisphere and rectilinearity in the PPA or RSC (both *F*_(1,7)_s < 2.5, *p*s > 0.16). In the OPA, there was a significant interaction between hemisphere and rectilinearity (*F*_(1,7)_ = 5.69, *p* < 0.05, $\eta_{p}^{2}$ = 0.45), with a numerically greater response to high- than low-rectilinearity in the left hemisphere, and the converse effect in the right hemisphere, although neither hemisphere exhibited a significant effect of rectilinearity (both *t*_(7)_s < 0.44, *p*s > 0.3). There was no interaction between category and hemisphere in any scene region (all *F*_(1,7)_s < 2.61, *p*s > 0.15).

Like the scene regions, EVC responded similarly to high and low rectilinearity stimuli (Figure 3A; *F*_(1,7)_ = 0.85, *p* = 0.39), and there was also no significant interaction between category and rectilinearity (*F*_(1,7)_ = 0.98, *p* = 0.36) or hemisphere and rectilinearity (*F*_(1,7)_ = 0.66, *p* = 0.44). Moreover, there was no significant region-by-rectilinearity interaction between EVC and any of the scene regions (all *F*_(1,7)_s < 2.52, *p*s > 0.18). However, EVC responded more to scenes than faces (*F*_(1,7)_ = 23.33, *p* < 0.01, $\eta_{p}^{2}$ = 0.77), and there was a significant interaction between hemisphere and category (*F*_(1,7)_ = 11.29, *p* < 0.05, $\eta_{p}^{2}$ = 0.62). Although PPA and OPA showed a greater scene-preferential response than EVC (all *F*_(1,7)_s > 39.95, *p*s < 0.001, $\eta_{p}^{2}$s > 0.85), the presence of category effects in EVC nonetheless indicates that there are some low-level differences between the scene and face categories despite our efforts to control for rectilinearity, overall visual extent, contrast, and luminance. There was no region-by-category interaction between EVC and RSC (*F*_(1,7)_ = 0.44, *p* = 0.53).

We considered the possibility that a subregion in the vicinity of the PPA, but not the most scene-selective part of the region, might be selective for the presence of right angles. Such a subregion might not be included in the PPA as defined by the top 100 voxels showing scene-selectivity in each hemisphere in the functional localizers. To address this possibility, we compared rectilinearity-selectivity (high vs. low rectilinearity contrast *t*-statistic) and category-selectivity (scene vs. face category contrast *t*-statistic) for each participant for each voxel in both hemispheres in the group-defined PPA parcel, which is larger than the individually-defined ROIs (Julian et al., 2012). Figure 3B shows the results of this comparison. Only a small fraction of voxels was more rectilinearity-selective than scene selective. Further, in each participant the number of category-selective voxels far exceeded the number of rectilinearity-selective voxels (Figure 3B). Thus, it is unlikely that the failure to find rectilinearity-selectivity in the PPA was due to a bias in ROI definition induced by our analysis methods.

**Figure 4:**
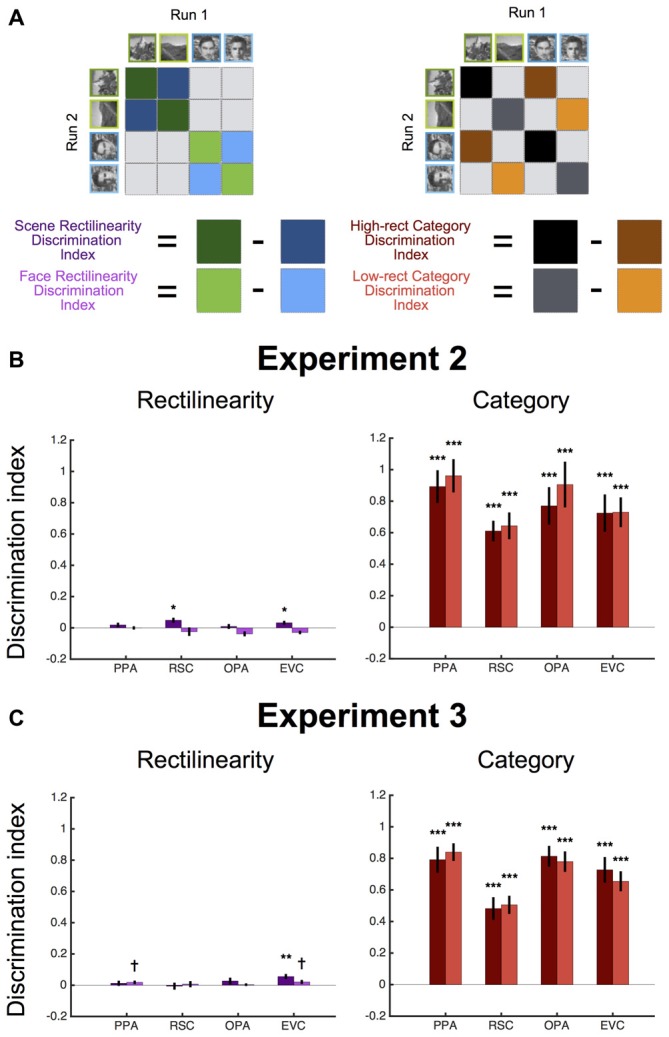
**Multivariate results for Experiments 2 and 3. (A)** To test for information about rectilinearity in each ROI, we computed a discrimination index that was the difference in split-half pattern similarity (r) between the same rectilinearity conditions and different rectilinearity conditions, separately for scenes and faces. To test for information about stimulus category, we computed a discrimination index that was the difference in split-half pattern similarity between the same category conditions and different category conditions, separately for high- and low-rectilinearity stimuli. **(B)** Experiment 2 average discrimination indices (±1 SEM) for rectilinearity and category in each ROI. There was no information about rectilinearity independent of category in any ROI, although all ROIs exhibited significant information about stimulus category.** (C)** Experiment 3 average discrimination indices (±1 SEM) for rectilinearity and category in each ROI. There was significant information about rectilinearity in early visual cortex (EVC), but not in other ROIs. All ROIs exhibited significant information about stimulus category. (^†^*p* < 0.08; *p < 0.05; **p < 0.01; ***p < 0.001).

We also considered the possibility that the PPA might be sensitive to rectilinearity at the representational level. That is, even though the overall level of activity in PPA did not distinguish between high vs. low rectilinearity scenes and faces, such distinctions might be apparent in multi-voxel response patterns. To test this, we performed split-half MVPA to see if it was possible to distinguish between stimuli based on rectilinearity (Figure 4A). We did not find strong evidence for this: in PPA and OPA, scenes with the same level of rectilinearity were no more representationally similar than scenes with different levels of rectilinearity (both *t*_(7)_s < 1.20, *p*s > 0.26), and an absence of rectilinearity information was also observed for faces (both *t*_(7)_s < −0.03, *p*s > 0.51). In RSC and EVC, there was significant information about rectilinearity for scenes (both *t*_(7)_s > 2.41, *p*s < 0.05), but not faces (both *t*_(7)_s < −0.82, *p*s > 0.78). In contrast, both high- and low-rectilinearity stimuli were more representationally similar if they were drawn from same category than if they were drawn from different categories in all ROIs (all *t*_(7)_s > 5.83, *p*s < 0.001; Figure 4B). These results reinforce the idea that scene regions are driven more by category than by rectilinearity.

In sum, our data in this case did not support the rectilinearity hypothesis. Not only was the categorical effect maintained after rectilinear matching, but no rectilinearity effect was observed for scenes and faces. Moreover, MVPA could not distinguish between stimuli that differed only in rectilinearity independent of category.

### Experiment 3: Does the PPA Exhibit a Rectilinearity Effect for Scenes and Faces with Artificially Enhanced Rectilinearity?

One possible reason for the lack of a rectilinearity effect in Experiment 2 may have been that difference between the high- and low-rectilinearity conditions was too subtle to be noticed by participants. Although the high- and low-rectilinearity conditions in Experiment 2 differed on rectilinearity according to the rectilinearity index designed by Nasr et al. (2014), this index may fail to capture the most perceptually salient rectilinearity dimensions. Further, the greater response to scenes than faces in EVC in Experiment 2 emphasizes that there were other uncontrolled low-level differences between the categories, complicating the interpretation of the category results. To address these concerns, participants in Experiment 3 viewed images of scenes and faces with artificially enhanced or degraded rectilinearity (Figure 1C). Images were decomposed into square pixels (pixelated) to increase rectilinearity or round points (pointillized) to decrease rectilinearity. We then examined the fMRI response in each of the predefined ROIs.

Once again, we failed to find an effect of rectilinearity on PPA response (Figure 3C). A 2 × 2 ANOVA with factors for category (scene vs. face) and rectilinearity (pixelated vs. pointillized) found greater response in the PPA to scenes compared to faces (*F*_(1,14)_ = 100.19, *p* < 0.0001, $\eta_{p}^{2}$ = 0.88) but no difference between pixelated and pointillized stimuli (*F*_(1,14)_ = 0.93, *p* = 0.35). This lack of a rectilinearity bias was found for both scenes (*t*_(14)_ = 1.20, *p* = 0.25) and faces (*t*_(14)_ = 0.20, *p* = 0.84). There was no interaction between category and rectilinearity (*F*_(1,14)_ = 0.46, *p* = 0.51). Further, comparing rectilinearity selectivity (pixelated vs. pointillized contrast *t*-statistic) and category selectivity (scene vs. face category contrast *t*-statistic) for each voxel in both hemispheres of the group-defined PPA parcel (Julian et al., 2012), there were few PPA voxels that were more selective for right angles than for scenes, and each participant exhibited substantially more voxels selective for scenes than right angles (Figure 3D). Thus, the failure to find rectilinearity-selectivity in the PPA in the present experiment was not due to a bias induced by our method of defining the ROI.

Results in the other scene regions were similar. Both RSC and OPA responded more to scenes than faces (RSC: *F*_(1,14)_ = 96.60, *p* < 0.001, $\eta_{p}^{2}$ = 0.87; OPA: *F*_(1,14)_ = 90.15, *p* < 0.0001, $\eta_{p}^{2}$ = 0.87), but neither region showed a significant main effect of rectilinearity (RSC: *F*_(1,14)_ = 1.02, *p* = 0.33; OPA: *F*_(1,14)_ = 2.96, *p* = 0.11), although the nonsignificant trend in OPA was in the predicted direction. There was no interaction between category and rectilinearity (RSC: *F*_(1,14)_ = 0.69, *p* = 0.42; OPA: *F*_(1,14)_ = 1.79, *p* = 0.20). Comparison of the three scene regions revealed no interaction between region and rectilinearity (*F*_(2,28)_ = 2.26, *p* = 0.12), but an interaction between region and category (*F*_(2,28)_ = 45.19, *p* < 0.001, $\eta_{p}^{2}$ = 0.78; scene-face response difference: OPA > PPA > RSC). EVC did not show effects of category (*F*_(1,14)_ = 1.39, *p* = 0.26) or rectilinearity (*F*_(1,14)_ = 0.38, *p* = 0.55), and no interaction between category and rectilinearity (*F*_(1,14)_ = 2.61, *p* = 0.13), indicating the category effects in scene regions could not have been inherited from EVC. There was no significant region-by-rectilinearity interaction between EVC and any of the scene regions (all *F*_(1,14)_s < 1.15, *p*s > 0.30), but all scene regions showed a greater scene-preferential response than EVC (all *F*_(1,14)_s > 12.50, *p*s < 0.004, $\eta_{p}^{2}$s > 0.47). 2 × 2 × 2 ANOVAs with hemisphere as a factor found no significant interactions between hemisphere and rectilinearity or category in any ROI (all *F*_(1,14)_s < 3.98, *p*s > 0.07).

To test whether the scene regions distinguished between pixilated and pointillized stimuli at the level of multivoxel patterns, we again performed split-half MVPA (Figure 4C). There was no significant information about rectilinearity in the RSC or OPA for either scenes or faces (both *t*_(14)_s < 1.22, *p*s > 0.12). The PPA showed marginal information about rectilinearity for faces (*t*_(14)_ = 1.59, *p* = 0.07, but not scenes (*t*_(14)_ = 0.72, *p* = 0.24). All ROIs contained significant information about category for both pixelated and pointilized stimuli (all *t*_(14)_s > 6.46, *p*s < 0.0001). Notably there was significant information about rectilinearity in EVC for scenes (*t*_(14)_ = 3.28, *p* < 0.01) and marginal information for faces (*t*_(14)_ = 1.52, *p* = 0.075), indicating that this region was sensitive to the difference between the pixilated and pointillized stimulus conditions.

### Effects of Category and Rectilinearity Outside of our ROIs

**Figure 5:**
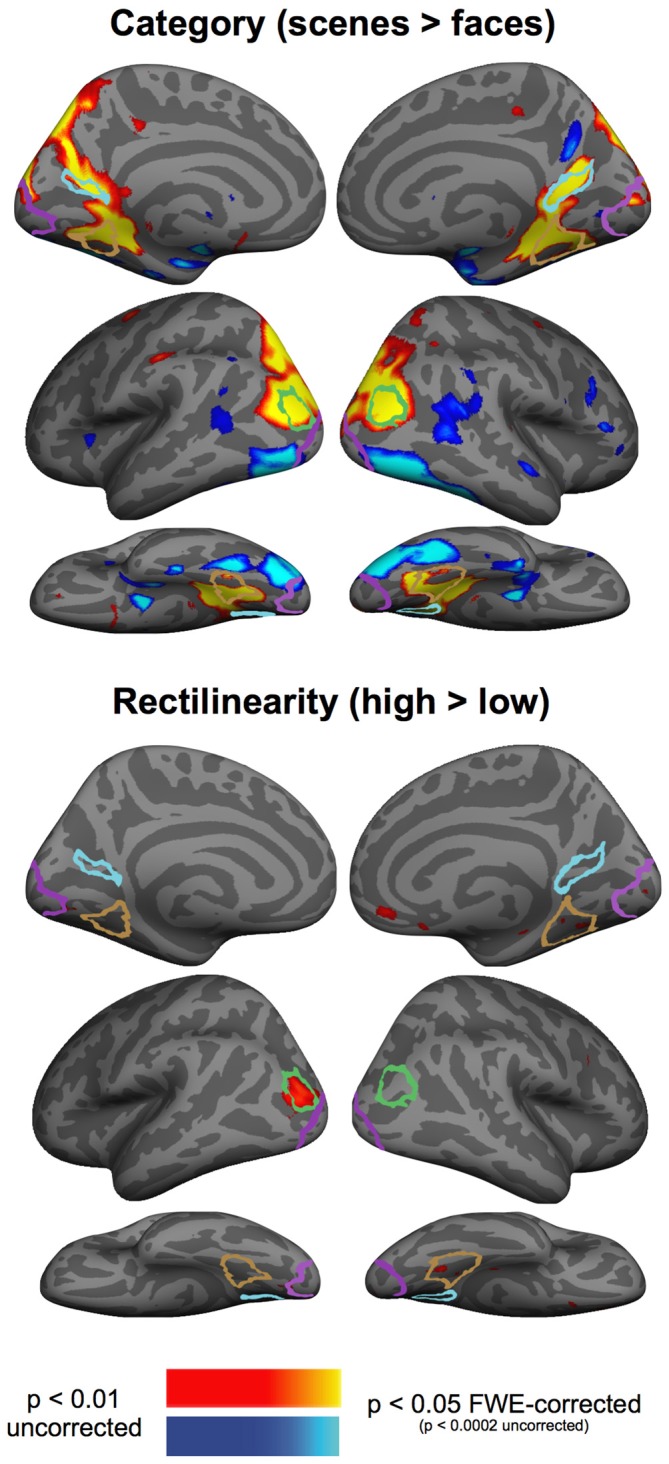
**Group-averaged contrast maps of the effect of category (scenes > faces contrast) and rectilinearity (high-rectilinearity > low-rectilinearity contrast).** Outlines of the group-defined parcels are shown for the PPA in brown, RSC in light blue, OPA in green, and EVC in purple.

To test for effects of rectilinearity and category outside of our ROIs, we performed a whole-brain group analysis, aggregating data from Experiments 2 and 3 to maximize power to detect effects of rectilinearity and category. This analysis revealed very strong category effects throughout high-level visual cortex (Figure 5): the PPA, RSC, and OPA responded more strongly to scenes, whereas lateral and ventral occipitotemporal regions responded more strongly to faces. By contrast, we observed no rectilinearity effects that survived correction for multiple comparisons, although notably there was sensitivity to rectilinearity observed near the posterior right PPA and the left OPA at uncorrected statistical thresholds.

## Discussion

Replicating the findings of Nasr et al. (2014), our data provide evidence that the PPA is sensitive to the presence of right-angle junctions in basic shapes (Experiment 1). This result suggests that rectilinearity may play a role in PPA stimulus tuning. However, the rectilinearity bias observed in Experiment 1 failed to explain PPA scene-selectivity in two further experiments. In particular, we did not observe a rectilinearity effect for naturalistic scene or face stimuli (Experiment 2), even when these stimuli had artificially enhanced rectilinearity (Experiment 3). Furthermore, the PPA responded more to scenes than faces in Experiment 2 even when the scene and face stimuli were matched on rectilinearity. The presence of a PPA rectilinearity bias in Experiment 1, but not Experiments 2 and 3, suggests that sensitivity to a single low-level image feature, namely right-angle junctions, may be more relevant to understanding PPA tuning to basic shapes than to naturalistic stimuli. More broadly, these results demonstrate that sensitivity to rectilinearity is insufficient to explain PPA category tuning.

Why might the PPA exhibit sensitivity to rectilinearity for basic shapes, but not naturalistic scene and face images? One possibility is that basic shapes are interpreted as being more scene-like when they contain more right angles. Scenes and faces, by contrast, do not suffer from such ambiguity of interpretation: they are clearly either scenes or non-scenes, irrespective of their rectilinearity content. Alternatively, although our naturalistic stimuli were matched on overall visual extent, luminance, and contrast, the high- and low-rectilinearity stimuli in the present studies may have differed along some other uncontrolled stimulus dimension that modulated the PPA response in the opposite direction of the predicted rectilinearity effect. Finally, the scene stimuli used in the current experiments only depicted naturalistic outdoor scenes, and it is possible that the PPA response is modulated by rectilinearity for other scene categories (e.g., man-made scenes; Walther and Shen, 2014). However, note that for these latter two possibilities, even if there were uncontrolled low-level differences between the stimulus conditions, and even if a rectilinearity effect were observed for man-made scenes, the lack of a rectilinearity effect in the current data is still evidence against the strongest form of the rectilinearity hypothesis (i.e., that the PPA response is determined mainly by stimulus rectilinearity).

Before dismissing the significance of right-angle junctions to PPA scene tuning, there is one caveat that merits mention. In the present study, rectilinearity of an image was calculated using an index introduced by Nasr et al. (2014). It is possible that the PPA is selective for rectilinear edges, but this rectilinearity index fails to detect the rectilinear edges to which the PPA is most tuned. In our experiments, there are at least three ways in which the index may have been inadequate. First, the rectilinearity index only reflects the presence of rectilinear edges at four spatial scales. While we found some insensitivity to spatial scale in Experiment 1, the PPA may be particularly sensitive to rectilinear edges at larger or smaller spatial scales than those in the current stimulus sets. Second, although this rectilinearity index is robust to rotations and translations of rectilinear edges in an image, it lacks invariance to skew deformations introduced by shifts in real-world viewpoint. The PPA could be highly sensitive to veridical rectilinearity—that is, true right angle junctions in the world—while maintaining invariance to the angular distortions caused by viewpoint shifts as they appear on the retina. If the PPA is tuned to this veridical (rather than image-level) rectilinearity, the current index would be sufficient from the vantage of a viewer positioned orthogonally to the surface plane, but not for a viewer positioned oblique to the plane, and the low-rectilinearity stimuli in the present experiments may have contained more right angle junctions oblique to the plane of the viewer. Finally, it is possible that the rectilinearity difference between the high- and low-rectilinearity conditions in Experiments 2 and 3 was smaller than in Experiment 1, or than in previous reports. Ideally, it would be possible to compare the rectilinearity range across stimulus sets. However, because the rectilinearity index normalizes rectilinearity within an image set, comparing across stimulus sets directly is problematic; in order to compare stimulus sets, rectilinearity values for each image must be recomputed, and this can cause changes in relative rectilinearity if new minimum or maximum rectilinearity values are introduced.

These caveats aside, we believe that our results serve to illustrate some of the possible dangers in attributing the responses of high-level visual areas such as the PPA to low-level biases. Granted, if the PPA and other scene-selective regions are involved in the perceptual analysis of the currently visible scene, it should be possible to explain how selectivity for scenes emerges from low-level representations in early visual areas (Op de Beeck et al., 2008). This observation does not imply, however, that scene region tuning can be reduced to a small set of visual features. There are four reasons for this. First, as discussed, low-level feature biases in the scene regions may occur simply because images with those features are more likely to be interpreted as scenes. Second, it is possible that scene-selectivity reflects tuning to the feature conjunctions that jointly define scenes, rather than a small set of low-level features. Third, representations in the scene regions may be tolerant to identity preserving transformations of the low-level features towards which these regions exhibit some bias (e.g., Marchette et al., 2015). Fourth and finally, the extent to which the scene regions are purely involved in visual perception rather than multimodal processing of scene shape and identity remains unknown (e.g., Wolbers et al., 2011).

Indeed, the issues addressed in the present work are germane to other debates regarding the mechanism of category-selectivity of other ventral visual stream regions. For instance, biases toward curvilinear shapes (Wilkinson et al., 2000; Caldara et al., 2006), increasing contrast (Yue et al., 2011), and the upper visual field (Caldara et al., 2006) have been reported in the face-selective fusiform face area (FFA). Findings such as these in the FFA have been taken to imply that low-level stimulus features may determine FFA tuning (Caldara et al., 2006; Yue et al., 2011). As with the scene regions, however, some caution must be taken here as well. For example, the FFA may be sensitive to curved shapes simply because such stimuli tend to look more face-like. In general, if some low-level feature is proposed to explain some brain region’s category selectivity, even in part, we propose a simple test, implemented here and inspired by previous approaches taken to understand FFA face-selectivity (Yue et al., 2011). Insofar as it is possible, it should be tested whether that region responds more to its preferred category than non-preferred categories when stimuli are matched on that low-level feature.

The results of the present experiments also reinforce the importance of testing whether low-level biases detected in high-level visual areas survive semantic variation in naturalistic image sets. We failed to find a rectilinearity bias in the PPA for natural scene and face images. In general, high-level regions may exhibit biases toward certain low-level image statistics for low-complexity stimuli because such low-level statistics are a defining characteristic of the preferred stimulus category (like sphericity for faces). Preferences observed in low-dimensionality image sets cannot thus be interpreted as sufficient explanations of cortical selectivity in general. For such claims, robust effects must be demonstrated across a wide range of semantic categories using naturalistic stimuli.

Finally, the types of information represented in the ventral visual stream may be sensitive to task demands. In the present work, participants performed a repetition detection task, whereas in Nasr et al. (2014) participants performed an orthogonal attention task that involved detecting if a small fixation point changed shape. The task in the present experiments may thus have required greater attention to stimulus identity than the orthogonal perceptual task employed by Nasr et al. (2014). The influence of low-level image statistics on activity in late visual areas may be exaggerated by orthogonal attention tasks. Indeed, task demands modulate representations across the ventral visual stream (Egner and Hirsch, 2005; Harel et al., 2014; Erez and Duncan, 2015), and in the PPA specifically attention has been shown to attenuate the processing of task-irrelevant background scenes (Yi et al., 2004). By directing attention to visual features unrelated to stimulus identity, the relative importance of low-level image properties in driving univariate responses may be inflated.

In sum, we found that rectilinearity is not sufficient to explain the category selectivity of the PPA. This result illustrates that reductive efforts to explain high-level semantic preferences with low-level image statistics, while informative, may not elucidate the ultimate mechanism of category-selectivity in high-level visual areas.

## Author Contributions

Conceptualization, PBB and JBJ; Methodology, PBB and JBJ; Software, PBB and JBJ; Formal Analysis, PBB and JBJ; Investigation, PBB and JBJ; Writing, PBB, JBJ and RAE; Visualization, PBB, JBJ, and RAE; Supervision, JBJ and RAE; Funding Acquisition, RAE.

## Funding

This work was supported by NIH (R01 EY-022350) and NSF (SBE-0541957) Grants to RAE and an NSF Graduate Research Fellowship to JBJ.

## Conflict of Interest Statement

The authors declare that the research was conducted in the absence of any commercial or financial relationships that could be construed as a potential conflict of interest.

## Footnotes

^1^http://fsl.fmrib.ox.ac.uk/fsl/fslwiki/

^2^http://surfer.nmr.mgh.harvard.edu/
